# Gamification as Online Teaching Strategy During COVID-19: A Mini-Review

**DOI:** 10.3389/fpsyg.2021.648552

**Published:** 2021-05-21

**Authors:** Francisco Antonio Nieto-Escamez, María Dolores Roldán-Tapia

**Affiliations:** ^1^Department of Psychology, University of Almería, Almería, Spain; ^2^Center for Neuropsychological Assessment and Neurorehabilitation (CERNEP), University of Almería, Almeria, Spain

**Keywords:** gamification, videogame, simulation, COVID-19, distance learning, motivation, student satisfaction, engagement

## Abstract

The ongoing pandemic caused by coronavirus disease 2019 (COVID-19) has enforced a shutdown of educative institutions of all levels, including high school and university students, and has forced educators and institutions to adapt teaching strategies in a hasty way. This work reviews the use of gamification-based teaching during the pandemic lockdown through a search in Scopus, PsycINFO, ERIC, and Semantic Scholar databases. A total of 11 papers from Chemistry, Business, Computer Science, Biology, and Medical areas have been identified and included in the present work. All of them analyzed the use of gamification strategies during the COVID-19 pandemic and assessed student’s learning and motivation outcomes. In general, students reported that gamification was innovative, engaging, and an efficient strategy to deliver curricula material; moreover, it was perceived as a fun activity. Some students reported that gamified videoconferences aided to connect with their classmates during isolation time providing effective social support. However, some students reported a bad physical or psychological condition, as consequence of the confinement, and did not get involved in the activity. Some weaknesses of the reviewed studies are the small sample size and its homogeneity, which makes it difficult to generalize their results to other scenarios and academic areas. Furthermore, although there is a feeling of learning during the activity, this result is mainly based on subjective perceptions, and any of the studies demonstrated that superior learning was achieved in comparison with traditional teaching strategies. Nevertheless, gamification can be implemented together with traditional lectures and can be a valuable instrument during post-COVID times.

## Introduction

In a time disrupted by coronavirus disease 2019 (COVID-19), the development of educational tools compatible with social distancing has become a fundamental strategy as millions of students are confined to reduce the spread of the epidemy. Thus, almost all teaching has quickly transitioned to distance education in order to provide appropriate social distancing (Johnson et al., 2020). Although social distancing has been accompanied by online interactions, it has been possible thanks to the continuous advances in digital technologies. Technology also gives the student much access to information and promotes the creation and sharing of knowledge, but it requires educators to work to find ways of increasing students’ motivation and engagement. Thus, a great amount of work has also been devoted to develop new teaching strategies that enhance students’ motivation and commitment and maximize their knowledge acquisition. Among different strategies, gamification has attracted the interest of educators, who in the last times have been exploring its potential to improve student learning (Dichev and Dicheva, 2017; Majuri et al., 2018; Koivisto and Hamari, 2019). Studies about the effectiveness of gamification are promising, with variable to positive results (Caponetto et al., 2014; Majuri et al., 2018; Osatuyi et al., 2018; Koivisto and Hamari, 2019).

Although “game” is an ambiguous term and different game formats have been used by researchers and educators (Hanghøj, 2013), gamification can be defined as the use of game elements in non-entertainment contexts to promote learning. The fact that games have many elements that are naturally appealing for young and adults and have a strong influence in their lifestyle helps to introduce an extra motivation for learning. Over the last decade, gamification is being increasingly employed in learning environments as a way to enhance students’ motivation and encourage social interaction. Thus, games have been employed in many educational contexts across different educational levels, showing its potential to improve learning outcomes (Seaborn and Fels, 2015; Koivisto and Hamari, 2019). The symbiosis between gaming and learning is also evidenced by the progressive development of best practices for courses gamification and game design. Usually, the game (course) is designed to progressively introduce new concepts to be mastered; students must then apply these concepts to increasingly challenging problems and ultimately apply prior knowledge to new situations (Varonis and Varonis, 2015). Another reason for including game elements in education is that it has been reported that games can provide social links (Waytz and Gray, 2018), promote knowledge seeking (Toh and Kirschner, 2020), develop creativity (Vartanian and Beatty, 2015), improve mental health (Cruea, 2020), and reduce isolation (Valkenburg and Peter, 2009).

There exist several types of games and gamification strategies. Quizzes are one of the simplest ways to gamify teaching, allowing students to test their knowledge on different platforms, such as web-based quizzes or apps. In recent years, educators have developed thousands of electronic quizzes as apps to assist students in many areas. Additionally, different strategies have been employed: the challenge-, the immersion-, and the social-based gamification. The first strategy is based on overcoming challenges (Majuri et al., 2018; Koivisto and Hamari, 2019). The second model attempts to immerse the user into a story and is characterized by its audiovisual richness (Concannon et al., 2019). Finally, social-based games permit to develop strategies of competition and collaboration (Romero, 2017).

Gamified activities have been linked to enhancing students’ intrinsic and extrinsic motivation. In this line, the self-determination theory places the focus on three basic psychological needs: autonomy, relatedness, and competence (Richter et al., 2015). Thus, gamification must fulfill at least one of them. Another theory that has been associated with gamification is the goal-setting theory. According to this theory, there are four factors linked to students’ performance: their commitment toward the goal, the feedback they receive, the complexity of the activity, and the situational constraints (Locke and Latham, 2002, 2006; Landers, 2014). According to this theory, gamification would require a challenge, an indication of progress, some feedback, levels of achievement, and a sort of competition (Huang and Hew, 2018). The third theory related to gamification is flow theory, where an optimal psychological and physical state maximizes enjoyment and engagement. According to this theory, gamification requires specific and understandable goals, immediate feedback, achievement indicators, and an adequate balance between challenges, student’s skills, and perceived value of the activity (Huang and Hew, 2018).

Thus, according to the goal-setting and flow theories, besides designing applications with capability to increase students’ motivation, teachers must also consider the special difficulties that their students face during confinement. Therefore, they can use gamification to mitigate physical and psychological constraints associated with a situation of quarantine. Furthermore, not all students have high-tech devices or appropriate internet connection at home, which restrict the generalized adoption of gamification for distance learning situations, as has been observed during the COVID-19 pandemic, particularly in developing countries and rural areas.

This work is aimed at reviewing the published experiences of gamified learning in secondary school and university education during the COVID-19 pandemic. We will describe the gamification strategy, the methodologies used during the COVID-19 pandemic, and its motivational and educational outcomes. Finally, we also pretend to analyze the theoretical base of these gamification strategies and how they have helped to ameliorate the situation of the students from a physical and psychological situation.

## Gamification Case Studies in COVID-19 Times

Although there exists previous evidence about the use of online tools and games in education, the number of studies using gamified strategies during the COVID-19 pandemic is scarce. There are, however, a large number of publications describing research proposals, protocols, and expert opinions regarding the implementation of digital tools in education. The sudden development of the COVID-19 outbreak made it difficult to plan empirical studies that tested the use of gamified tools, with the majority of educators doing huge efforts to move from a face-to-face classroom environment to online lectures through videoconferencing tools.

In the present work, a non-systematic search for terms included in the title, abstract, or keywords using the following syntax [(“distance” OR “remote”) AND (“teaching” OR “learning” OR “education”) AND (“covid” OR “pandemic”) AND “gam^∗^”] has been carried out. The search was done on February 28, 2021 for studies published between January 2020 and February 2021 in Scopus (46 results), PsycINFO (2 results), ERIC (1 result), and Semantic Scholar (1,450 results) databases. Only research articles and conference papers describing a gamified practice in a learning environment have been included. Studies not written in English, reviews, surveys, and opinion papers that did not carry out any gamified practice have been excluded. A total of 11 studies that met these eligibility criteria have been included and reviewed here. The studies reviewed correspond to the following areas of knowledge: Chemistry, Business, Computer Science, Biology, and Medical education. A summary of the reviewed studies is shown in Table 1.

**TABLE 1:** Articles included in the review: objectives, methodology, and outcomes.

Article	Gamification objectives	Game elements	Data collection	Motivational outcomes	Learning results
da Silva et al. (2020a)	To help students review concepts in a collaborative environment	Competition, cooperation, challenge, points, and leaderboard	Exam scores, student feedback through questionnaires	Increased motivation through competition for leaderboard rank	Learning improves in the same way as regular problem-solving classes
Fontana (2020)	To maintain students’ wellness and class community and to develop knowledge	Competition, points, and videoconference	Student feedback through surveys, observation of students’ behavior	Increased engagement, improved class morale, and enthusiasm	Subjective perception of learning
Pearson (2020)	To provide a remote revision aid tool	Puzzle (no immediate feedback	Student-tracking statistics, cohort exam scores, and student feedback questionnaire	Increased confidence and increased engagement in some students	Higher percentage of students scoring over 75 and 87.5%
D’Angelo (2020)	To reinforce laboratory topics and to engage students	Escape room-based procedure	Student-tracking statistics	No real engagement	Bad performance in general
Pakinee and Puritat (2021)	To motivate and engage students	Avatars, challenges, points, levels, progress, and leaderboards	Pre–post-exam, student-tracking statistics, and interviews	Higher engagement but short lasting	No differences between gamified and non-gamified groups
Lelli et al. (2020)	To motivate and engage students and to review and practice theoretical knowledge	Avatars, points, levels, and missions	Tasks scores, forum comments, questionnaire responses, list resolution, quiz, and participation in debates	Remote activities were not mandatory and participation was lower than expected	Subjective and variable perception of learning in a reduced number of participants
Liénardy and Donnet (2020)	Theory concepts reminder	GameBook	Exercises performance	Low participation	Not assessed
Lobet et al. (2020)	To learn biological vocabulary and to enhance motivation	Treasure hunt, photo quiz	Picture collection and photo quiz accuracy, student feedback survey	Higher engagement in hunt than in photo quiz activity	Feeling of having learned. Lower accuracy in photo quiz
O’Connell et al. (2020)	To review of core obstetrics and gynecology topics	Imaging quiz, points, and competition	Responses to quiz questions, assessment of proposal for case management, and post-session survey	95% showed engagement	High feeling of having learned
Kobner et al. (2020)	Development of clinical reasoning skills and to improve engagement	Serial cues, simulation, dice, and videoconference	Semi-structured interviews	Increased engagement	Feeling of improved clinical reasoning abilities
Patel et al. (2020)	To enhance medical knowledge	Simulation, videoconference	Pre- and post-test, and final survey	Limited engagement due to larger group size	Statistically significant knowledge gain

### Chemistry Learning

Organic Chemistry is considered a difficult subject for secondary school and undergraduate students, being organic reactions are one of the most difficult topics in Organic Chemistry (Eticha and Ochonogor, 2015). Previous studies show that when learning activities of chemistry concepts are combined with games in the classroom, students’ motivation increases (Stringfield and Kramer, 2014) improving their performance (Liberatore, 2011; Revell, 2014) and making them more engaged compared with traditional methodologies (Sousa Lima et al., 2019; da Silva et al., 2020b).

da Silva et al. (2020a) designed an interactive game-based application (Interactions 500) aimed to help students review concepts related to intermolecular forces in a collaborative environment. This game was originally designed to be used by students in the classroom; however, the interruption of face-to-face classes due to the COVID-19 pandemic required to use it remotely. Forty-four pharmacy undergraduate students (11 groups) played the game remotely. A student got the role of a leader who was in charge of coordinating the game, motivating their mates, discussing answers, and clarifying doubts. In the game, there was a competition among students who had to answer different quiz questions. The students rated the game very positively through a Likert-type survey with regard to its design, content, game play, and usefulness as educational tool. The authors compared the knowledge of a group of 44 students who played the game with regard to another group (N = 40) who were not exposed to it and studied alone at home. Both groups showed similar marks in the pretest and the post-test and the same level of learning. Therefore, the authors concluded that the game resulted in similar learning outcomes to traditional problem-solving classes, although only the game created a pleasant learning environment, so all the students who played the game reported that they preferred it with regard to regular problem-solving classes.

Fontana (2020) developed a gamified activity based on ChemDraw (a software designed for drawing molecules) with the aim of making students get practice using this tool. Thus, students had to compete in a tournament. The idea was that it would maintain the classroom community, improve students’ wellness, and develop their organic chemistry skills. Videoconferencing software (Zoom) was used to enable real-time classroom participation. Nine students participated in the molecule speed-drawing tournament (Molecule Madness). A molecule’s chemical structure was posted to the class learning platform, and for each match, two students had to share their screens with the class and compete to correctly draw its structure first in ChemDraw. Non-participating students followed the tournament as active observers (social spectators), socially engaging with fellow observers and learning from contenders. Students competed to correctly draw molecular chemical structure, where advanced rounds presented molecules progressively more difficult to draw. By playing ChemDraw, students reported wellness experiences comparable with playing traditional videogames: enjoyed practicing, felt expectancy for the coming class activity, and connected with their classmates. They also described Molecule Madness as a fun way to learn organic chemistry, practice ChemDraw, and promote high levels of excitement and engagement. According to the author, postpandemic chemistry education will likely include some distance gaming elements that will enhance face-to-face teaching.

Chemistry crosswords have been used for a long time, and their effectiveness has been described as a tool for leaning during the COVID-19 pandemic. Pearson (2020) used crossword puzzles as a model of remote active learning for first- and second-year undergraduate pharmacy students. Chemistry-themed crossword puzzles were delivered *via* the eBlackboard site and used to supplement lectures and problems content. This activity started before the lockdown, so the author compared students’ behavior before and during the lockdown, with no clear differences between both periods. When analyzing students’ exam performance with and without crossword aids, no significant differences were observed in the mean and median cohort exam grades (compared with a cohort from the previous academic year). However, more students responded to the question (from a choice of four) taught alongside online crossword exercises. The author suggested that the crossword activity instilled greater confidence to answer a question when it had been included in the crossword exercises. Moreover, a larger percentage of students got higher marks in their exam after crossword exposure compared with the previous academic year, in the absence of the crosswords. The author suggested that the crossword impacted the exam performance for at least more engaged students. Around 50% of first-year students and 80% of second-year students reported that this activity was helpful and would welcome more. Moreover, in an online survey, 20.4% of the students rated quizzes and puzzles as the best remote teaching tool second only to instructional videos (46.3%).

The author proposed that, looking ahead, these puzzles should be delivered in a more interactive online format and provide instant feedback. Moreover, for optimum student engagement and learning improvement, instructors could design crosswords that help students identify key topics and concepts. Finally, another approach would be that the students create their own crosswords.

The COVID-19 pandemic learning disruption has seriously affected interactive and hands-on experiences in laboratories. Thus, D’Angelo (2020) developed a series of five exercises, called “Labventures,” mimicking the principle of “Choose your own adventure” books or escape rooms. The exercises were created to review/reinforce several tasks, and 24 students took part. Labventures stories were set up as a series of webpages, and the students should complete a laboratory task choosing proper techniques. After every incorrect response, the students were given feedback explaining why a choice was wrong, whereas correct choices moved the activity forward. However, the analysis of students’ execution indicated a low performance and understanding of the activities. The author proposed some improvements for future versions of these exercises, including pictures or videos, providing more data to encourage problem solving, using a notebook quiz, and giving further information after wrong choices.

In all the works, a small number of participants with reduced heterogeneity of the samples were included. In the case of da Silva et al. (2021), 44 pharmacy undergraduate students were included. In the studies done by D’Angelo (2020) and Fontana (2020), all the students belonged to the same classroom, whereas 132 first-year and 120 second-year undergraduate pharmacy students participated in the study of Pearson (2020). Nevertheless, the main weakness comes from the predominant use of subjective procedures to assess learning outcomes.

### Business Studies

Pakinee and Puritat (2021) investigated the effect of gamified and non-gamified learning for an Enterprise Resource Planning (ERP) course to motivate the students to engage and participate during working from home. Thus, two versions of an e-learning platform were developed, one implementing game elements (avatars, challenges, levels, points, progress bar, and leaderboard) and another one without game features (just the exams and course materials). Furthermore, in their study, the authors considered students’ personality traits (Extraversion, Neuroticism, Conscientiousness, Agreeableness, and Imagination/Openness). A test before and after each chapter and web monitoring of students’ activity provided quantitative data about their performance. According to the results, the gamified e-learning group showed higher engagement during the first 2 weeks, but then it started to drop, and at the fourth week, both groups had equaled their activity. The authors referred that the gamification strategy increased students’ activity in the short term but not in the long term. Another activity record showed that gamification motivated students to start working during the first days a lesson was available, whereas the non-gamified group delayed their activity to the end of the week. Nevertheless, there was no statistical evidence to support any differences in learning between the non-gamified and gamified groups.

According to the authors, gamification cannot improve the overall knowledge because it has positive and negative impacts on each personality type. Thus, some personality traits were linked to higher scores for the gamified e-learning because they are more prone to competition, whereas others reported a negative effect from competition. The authors affirmed that gamification in the ERP course can improve students’ motivation, although the fun and curiousness related to gamification are short-lived. They recommend adding new small tasks of game elements every 2–3 weeks until the end of the course, concluding that gamification of e-learning alone serves as a tool to engage students in distance learning, but in order to enhance learning, the presence of a lecturer is also required.

This work has the merit of including students’ personality traits as a research variable. Thus, it can provide some cues about how to design more personalized gamification strategies according to participant’s personality. Furthermore, it has compared the efficiency of gamified and non-gamified options. However, it has some limitations, for instance, the research was conducted only in one course (ERP) and the limited number of participants per group.

### Computer Science Learning

Lelli et al. (2020) addressed the concept of Emergency Remote Teaching (ERT) as a temporary shift of teaching in crisis circumstances and involves the use of fully remote teaching solutions. The authors described a gamification methodology for two Computer Science courses by using ClassCraft, a free educational platform from Google that works as a virtual classroom. This platform allows the teacher to assign tasks to the students and enables the use of asynchronous (forums, videos) and synchronous tools (chats) and a score based on the accomplishment of the tasks. A number of modules (missions) with different tasks were defined according to the courses content, and game levels for each module were set as students progressed through tasks.

The authors stated that the use of this gamification tool was effective to engage the students during the pandemic. Some students pointed out gamification as a positive experience to learn remotely. However, the number of students who participated in the remote activities decreased after some time, with some students reporting no physical and psychological conditions or interest in following the gamification activities. The authors also observed that students had difficulties to understand the purpose of using asynchronous tools, such as forums.

In another work, Liénardy and Donnet (2020) provided to first-year Computer Science students a set of gamified homework exercises, they called GameCode, aimed at teaching an appropriate methodology for programming. The exercises were inspired by GameBooks in which the reader can choose the path to complete the story. Students could choose their own solving path for each exercise and do it at their own pace. Any GameCode exercise met the following requirements: (a) each exercise was self-sufficient and contained the minimal information to complete it; (b) theoretical reminders were needed and were as short as possible; (c) hints never revealed the solution, nor a part of it; and (d) several solutions were always possible and could be discussed in the course forum. However, the authors reported that few students took part in the exercises, as many students had abandoned their courses, supposedly a consequence of the loss of motivation by the COVID-19 lockdown. Half of students who did the activity informed that they liked it, but 43% declared that they would have preferred podcasts.

In both studies, the reduced number of participants can be considered a serious weakness. In the study by Lelli et al. (2020), the use of a free platform will allow other researchers to replicate the same protocol in different courses. However, in both cases, the participation was lower than expected, which demonstrates a lack of motivation and engagement. Similar outcomes were obtained by Liénardy and Donnet (2020). In both cases, the authors explained the low participation as a consequence of the physical and psychological effects of COVID-19 confinement.

### Biology Studies

Teaching biology is particularly challenging if the students are not allowed to access laboratories for hands-on observation of fresh specimens and the lockdown restricted movement outside students’ home. Lobet et al. (2020) developed a biological treasure hunt activity for 346 first-year biology students by using QuoVidi, an open-source web-based platform. This platform was conceived to teach biological vocabulary and to observe the surrounding natural world. Students received a list of quests that addressed botanical and zoological terms. Students should understand the meaning of the quest and go out to get photos of plants and animals that should be uploaded to the platform. Due to movement limitations during the lockdown, there was the option of learning from photos submitted by other students (photography quiz). In this case, they had to match the submitted photos with their quest. Students showed a good performance as the majority of pictures submitted in the treasure hunt activity were correct. Nevertheless, performance was less accurate for the photography quiz, probably due to different levels of engagement. Regarding students’ feedback, 91% reported to like the activity and have learned from it, although there were two main criticisms, that the activity took so much time and some students had the feeling that they did not truly learn. The authors addressed these points by proposing a better tailoring of the activity and a better communication with the students about the pedagogical goal of the activity.

One strong point of this activity is its scalability, so hundreds of students can be involved. Furthermore, according to students’ feedback and performance, it was motivating and engaging in learning a list of technical vocabulary. Nevertheless, the photography quiz, included as a response to the lockdown, resulted in a worse performance compared with the hunting activity. Thus, it would be essential to redesign the photography quiz in order to make it more engaging and efficient.

### Medical Education

COVID-19 has challenged medical educators on continuing to provide quality educational content. O’Connell et al. (2020) described a novel virtual game for obstetrics and gynecology teaching. The game consisted of several rounds of rapid-fire questions and cases, eliminating teams to a final contest. All residents participated individually in a previous “warm-up” round, using a Kahoot quiz to test their knowledge of ultrasound imaging. The residents were divided into small groups and placed into a breakout room with a faculty facilitator who then divided their residents into two teams of three to four residents. The two teams then competed in the breakout room for the first three rounds. Each round focused on testing the team’s knowledge of a different aspect of obstetric and gynecological care. The fourth and final round was a series of three cases in which the remaining teams were given a case and they had to write down their proposal for how each case should be managed. The fourth round was judged by the faculty facilitators. At the end of the game, 23 out of the 36 residents completed an anonymous online survey. A large majority of the residents enjoyed the activity. Ninety-five percent of the residents were in agreement or strong agreement that they were engaged during the activity. Seventy-four percent were in agreement or strongly agreed that this activity was better than traditional lectures. Therefore, the majority of the residents found this activity to be educational, entertaining, engaging, and better than the traditional lecture format.

Medical students usually learn clinical reasoning through “whole-case” conferences. However, this procedure has many challenges in social distancing scenarios. Thus, although videoconferencing tools allow some interaction, audience engagement and active participation are limited. Kobner et al. (2020) have described a novel case conference format to train clinical reasoning skills to a spatially distant audience. In their work, the authors describe a gamified serial-cue, low fidelity simulation in which a team of residents must analyze a real case that challenges their clinical reasoning skills. The case includes all relevant diagnostic results, including several elements that challenge clinical reasoning abilities. The team of residents plays through a simulated tabletop version of the case live on a videoconference call. The case flow is facilitated by a chief resident familiar with serial-cue tabletop simulations and gaming procedures. A simulated cardiac monitor provides vital signs to the team and the virtual audience, and as team members ask for diagnostic studies, the facilitator provides them through Dropbox to the team and the audience. At the conclusion of the case, a debriefing was conducted. After this, a spontaneous discussion ensued, covering themes ranging from diagnostic decision to patient safety and foundational medical knowledge. Finally, a sample of simulation participants, the virtual audience, and residency program administrators were interviewed. All simulation participants felt that the tabletop simulation improved their clinical reasoning abilities in ways that mimicked real clinical encounters. They reported that the level of unpredictability helped to model the actual practice of emergency medicine, adding a level of excitement absent from typical mock oral boards-type tabletop simulations. Audience members agreed that they were more engaged throughout the case simulation than during traditional case conferences. Residency program administrators noted increased faculty engagement and discussion when compared with traditional case conferences. Finally, interviewees suggested that this experience would benefit from more gamification throughout the simulation.

Telesimulation can be employed to deliver hands-on training that usually takes place in in-person simulation. In order to assess if it can be effective to teach anesthesiology trainees to manage a complex case-based scenario, Patel et al. (2020) developed a remote high-fidelity immersive case-based scenario for anesthesiology residents training. For this, the authors adapted an existing simulation scenario based on a real clinical case. Fifty-eight residents were scheduled to participate remotely *via* Zoom meetings. For each session, a group of 6–8 residents participated in the simulation (a total of 8 sessions were carried out), whereas 4 faculty anesthesiologists were present in the simulated operating room with a manikin. Using Zoom’s share screen feature, images of the operating room and a manikin vital signs were monitored. The residents were asked to respond to the scenario and verbalize all the actions that they would perform in a real-life situation. An anesthesiologist present in the operating room performed actions based only on instructions from the residents’ team. Just before and after the simulation, participants’ medical knowledge was assessed through an online exam, and a satisfaction survey was conducted at the end of the activity.

Overall, telesimulation resulted to be effective at increasing residents’ knowledge as their score was superior in the post-test. They also rated the experience positively and informed that it could be a reasonable substitute for in-person learning. Nevertheless, the authors pointed out the importance of using small group sizes (3–5 students are the typical number for traditional simulations), assigning roles to participants, and using intermittent reflective pauses.

The main weakness of these studies is the small number of participants, and that they are based on a single-center study. Moreover, only students’ attitude was evaluated in O’Connell et al. (2020) and Kobner et al. (2020), and there are no results about the educational efficacy of the activity. Patel et al. (2020) conducted pre- and post-assessment of residents’ medical knowledge; however, there was no comparison for knowledge gains in telesimulation versus traditional simulation setups.

## Conclusion

In recent years, several innovations have emerged in the field of education. In an age disrupted by COVID-19, the development of gamified teaching strategies can be seen as a promising option to provide knowledge and enhance students’ collaboration during social distancing. Thus, although traditional scholarly academic curricula are content-focused and essentially ignore personal development, some gamification literature suggests that collaborative activities can stimulate motivation and enhance learning (Rutledge et al., 2018). All the studies described here aimed at enhancing learning by improving participants’ motivation and engagement. Some studies have used a pre-existing platform that has been gamified, but there are also some experiences in which a gamified application has been developed on purpose by the authors. In most of the cases, the gamified activity was well-received by learners, considered effective, educational, and engaging, and in some cases also fun.

One of the findings of this review is that most of the gamification experiences have been developed in Science, Technology, Engineering, and Mathematics (STEM) disciplines. This could be the consequence of the difficulties to carry out laboratory and hands-on practices during the COVID-19 pandemic, which particularly have affected these fields. Nevertheless, the efforts to introduce teaching innovations that help to overcome social distancing shortcomings have led teachers to improvise activities along the way. In many cases, this has been associated with a poor planning of the gamified environment, together with the *ad hoc* use of gaming elements mechanics with unclear guidelines for students. Thus, some of the studies included in the review (D’Angelo, 2020; Lelli et al., 2020; Liénardy and Donnet, 2020) showed little or no participation by students. According to the authors, such apathy would be associated with a decreased intrinsic motivation related to the pandemic situation. At least in these cases, the employed gamification strategy did not result in efficient tool for engaging students.

Although any of the reviewed studies have implemented a theoretical framework behind their gamification strategy, most of them have reported an increase of learning and/or motivation. Game elements associated with competition, such as leaderboards and points, have been the most common ones, resulting in higher levels of engagement and learning outcomes, with similar results being reported in simulation procedures and quizzes. Only one study used puzzles, reporting moderate results, whereas those that employed escape rooms and gamebooks resulted in negative outcomes.

It has been stated that competition elements affect extrinsic motivation in students mainly, without increasing intrinsic motivation (Erdogdu and Karatas, 2016). However, most of the reviewed studies have reported a sense of enjoyment and positive feelings toward learning, which are directly related to intrinsic motivation (Bai et al., 2020). Thus, all the reviewed studies that reported positive emotions associated with intrinsic motivation, even when external elements were employed (competition elements), referred to increased motivation, engagement, and/or learning outcomes. Thus, intrinsic and extrinsic motivation would correlate and show common properties as stated by the Self-Determination Theory (Ryan and Deci, 2002). However, some studies (D’Angelo, 2020; Lelli et al., 2020; Liénardy and Donnet, 2020) reported low motivation, engagement, and/or bad performance. In order to increase the interest and motivation of students, they should receive continuous support from the teaching staff, and the aim of the activity should be clear for all them.

Gamification procedures allowed to monitor students learning progress in a non-invasive way, for instance, tracking students’ behavior in the web platform, or their achievements in the game. Other strategies to gather information about students’ perception of their own progress relied on the use of questionnaires and interviews. However, some works have attempted to assess students’ learning through their performance in exams. In general, the reviewed studies have combined quantitative and mixed methods to assess students’ learning and engagement (see Table 1). In general, most of the reviewed works came to the conclusion that gamification resulted in learning outcomes. In some cases, this statement was a subjective perception of participants obtained through questionnaires, whereas other works performed objective tests to assess students’ knowledge. For instance, da Silva et al. (2020a), Pakinee and Puritat (2021), and Patel et al. (2020) compared students’ performance in pre- and post-exams, whereas Pearson (2020) compared exam scores with regard to previous year scores, showing an increase of students’ knowledge following gamified activities. However, there is no evidence indicating that gamification yields better learning outcomes than could be obtained with more traditional strategies. Furthermore, one study (Pakinee and Puritat, 2021) reported that gaming elements linked to competition resulted in controversy and did not produce the same effect in all students, and in some cases, they could increase or decrease motivation according to the student’s personality.

Social interaction is considered one of the foundations of gamification (Sánchez-Martín et al., 2017). All the studies were conducted in a time of limited social interaction; however, only Fontana (2020) had as a main goal to enhance students’ well-being and promote social interaction. The author reported an increase of class community, showing that the gamification strategy was useful to keep class morale during social distancing time.

According to the works reviewed, it is possible to infer that gamification can be effectively combined with traditional teaching methods, such as online lectures, in order to enhance students’ engagement and deliver curricula material that usually is taught through face-to-face education. Likely, technology-enhanced learning initiatives will become more prominent as the education landscape is reorganized following COVID-19, and gamification may therefore be considered as an option to augment traditional learning no longer deliverable at traditional face-to-face classes. It can be also incorporated into academic programs currently limited to videoconference lectures to boost students’ engagement and motivation.

There are, however, some weaknesses that must be taken into account. The reviewed studies are mainly a single-center study with data from single classroom groups, resulting in a relatively low number of participants, which restricts the generalization of their results. Furthermore, all studies included had a short-term format. Thus, longitudinal studies are required to determine the efficacy of gamification as teaching strategy. Nevertheless, the main limitations are the lack of an objective assessment of learning as result of the gamified activity and the lack of a theoretical framework. Although some studies (Lobet et al., 2020; Patel et al., 2020) reported that the students’ knowledge improved after the activity, there was no direct comparison with conventional teaching scenarios. Only Pakinee and Puritat (2021) compared the performance of their students in both situations, showing that gamification made students start working earlier, but there were no differences on their performance in the long term. This could indicate that gamification can open a more efficient time window during online learning. Therefore, the main conclusions of the reviewed studies are based on the subjective perception from participants: “a fun way to learn” or “feeling to have learned.” In some cases, this drawback resulted from the sudden lockdown imposed by the authorities, so teachers had to design and adapt their courses along the way.

We must remark that during the COVID-19 lockdown, many students faced increased demands at home, many had to bring together their studies with their job activities, caring for children during the day, along with an increase of academic online activities. Furthermore, some students and teachers could be resistant to implement a game as an educational tool as it is a new way of learning and teaching quite different from the traditional classes. It is also important that there is a good communication between teachers and students, so the pedagogical aim of the activity becomes clear.

Finally, it is important to bear in mind that there are many examples of trendy “gamechangers” in education that have varied greatly over time. Problem-based learning, for instance, once a main educational strategy in some curricula, and social media-based learning have lost part of their interest after a time of apogee (Guckian and Spencer, 2018). It is essential that educational innovations have a solid foundation on research data. In the case of gamification as an educational strategy, future research must address different aspects, such as game mechanics and elements, in relation to an underlying theoretical framework.

## Author Contributions

FN-E and MR-T conceived the present work, did the literature search, wrote the manuscript, and revised and verified the final version. Both authors have read and accepted the content of the manuscript.

## Conflict of Interest

The authors declare that the research was conducted in the absence of any commercial or financial relationships that could be construed as a potential conflict of interest.
